# Functional analysis of *Brassica napus* phloem protein and ribonucleoprotein complexes

**DOI:** 10.1111/nph.14405

**Published:** 2017-01-04

**Authors:** Anna Ostendorp, Steffen Pahlow, Lena Krüßel, Patrizia Hanhart, Marcel Y. Garbe, Jennifer Deke, Patrick Giavalisco, Julia Kehr

**Affiliations:** ^1^ Molecular Plant Genetics University Hamburg Biocenter Klein Flottbek, Ohnhorststr. 18 Hamburg 22609 Germany; ^2^ Max Planck Institute of Molecular Plant Physiology am Mühlenberg 1 Potsdam 14476 Germany

**Keywords:** blue native PAGE, *Brassica napus*, mass spectrometry, phloem, proteasome‐mediated protein degradation, protein complex, ribonucleoprotein complex

## Abstract

- Phloem sap contains a large number of macromolecules, including proteins and RNAs from different classes. Proteome analyses of phloem samples from different plant species under denaturing conditions identified hundreds of proteins potentially involved in diverse processes. Surprisingly, these studies also found a significant number of ribosomal and proteasomal proteins. This led to the suggestion that active ribosome and proteasome complexes might be present in the phloem, challenging the paradigm that protein synthesis and turnover are absent from the enucleate sieve elements of angiosperms. However, the existence of such complexes has as yet not been demonstrated.
- In this study we used three‐dimensional gel electrophoresis to separate several protein complexes from native phloem sap from *Brassica napus*. Matrix‐assisted laser desorption ionization‐time of flight MS analyses identified more than 100 proteins in the three major protein‐containing complexes.
- All three complexes contained proteins belonging to different ribosomal fragments and blue native northern blot confirmed the existence of ribonucleoprotein complexes. In addition, one complex contained proteasome components and further functional analyses confirmed activity of a proteasomal degradation pathway and showed a large number of ubiquitinated phloem proteins.
- Our results suggest specialized roles for ubiquitin modification and proteasome‐mediated degradation in the phloem.

## Introduction

The phloem is the major route for the transport of assimilates synthesized during photosynthesis. However, it is meanwhile well accepted that besides photoassimilates, other metabolites and phytohormones, also macromolecules such as proteins and RNAs can traffic through the phloem long‐distance system to distant plant parts (Hall & Baker, 1972; Lucas *et al*., 2001; Yoo *et al*., 2004; Buhtz *et al*., 2008, 2010; Kehr, 2012; Turnbull & Lopez‐Cobollo, 2013). The phloem system consists of companion cells (CCs), sieve elements (SEs), fibres, ray cells and phloem parenchyma cells. SEs and CCs, forming the conductive SE–CC complex, are the result of an unequal division of a common mother cell and remain connected by special secondary plasmodesmata (PD), called pore‐plasmodesm units (PPUs) (van Bel, 2003), during their complete lifetime. During differentiation, SEs lose important cellular components such as nuclei and functional ribosomes (Esau, 1969). Therefore, it is generally believed that mature SEs have lost the capability for transcription and translation and it has been observed that phloem sap components can even inhibit translation (Zhang *et al*., 2009). Therefore, the macromolecules found in phloem sap are probably synthesized in CCs and imported into SEs, as has first been demonstrated for the prominent phloem proteins PP1 and PP2 (Bostwick *et al*., 1992; Clark *et al*., 1997). Loading of molecules into the phloem is limited by the size exclusion limit (SEL) of the PPUs connecting CCs to SEs. The SEL can depend on plant species, tissue and phloem type, and developmental stage. An SEL between 10 and 40 kDa could be observed when fluorescently labelled compounds were injected into the stem phloem of broad bean (Kempers & van Bel, 1997). Overexpression experiments of green fluorescent protein (GFP) and GFP‐fusion proteins in Arabidopsis CCs suggest an SEL even higher than 67 kDa (Imlau *et al*., 1999; Stadler *et al*., 2005).

One major class of macromolecules found within phloem sap are polypeptides. Hundreds of them have already been identified in phloem sap samples from different plant species (Walz *et al*., 2002, 2004; Giavalisco *et al*., 2006; Lin *et al*., 2009). Many of these proteins could be classified as redox‐related proteins and many could potentially be involved in stress responses or signal transduction. Furthermore, many potential nucleic acid binding proteins could be observed that might play a role in RNA transport (Barnes *et al*., 2004; Giavalisco *et al*., 2006; Lin *et al*., 2009). In addition to proteins, a wide set of RNAs including messenger RNAs (mRNAs), transfer RNAs (tRNAs), ribosomal RNAs (rRNAs) and small RNAs (smRNAs) are common macromolecular components of phloem sap from different species (Yoo *et al*., 2004; Omid *et al*., 2007; Buhtz *et al*., 2008, 2010; Zhang *et al*., 2009; Kanehira *et al*., 2010; Thieme *et al*., 2015).

Some of the mobile phloem macromolecules probably form an important component of the communication network in higher plants. Specific mRNAs and smRNAs have been shown to act as phloem‐mobile signalling molecules during development and nutrient deficiency responses (Kim *et al*., 2001; Haywood *et al*., 2005; Buhtz *et al*., 2008, 2010; Pant *et al*., 2008; Martin *et al*., 2009). Signal transduction via RNAs and proteins is achieved via import into the phloem SEs and their transport to their target tissues.

It has been demonstrated that phloem proteins can interact with each other and with RNAs and that multiprotein and ribonucleoprotein complexes can be formed *in vitro* (Gomez & Pallas, 2004; Yoo *et al*., 2004; Gomez *et al*., 2005; Ham *et al*., 2009; Lin *et al*., 2009; Ma *et al*., 2010). However, none of the studies showed the existence of functional complexes present in native phloem samples. Therefore, we addressed the question of whether the observed interactions are transient or if stable high‐molecular‐weight complexes are present.

To identify and characterize high‐molecular‐weight protein–protein and ribonucleoprotein complexes, phloem sap from oilseed rape (*Brassica napus*) was sampled and subjected to a combination of three‐dimensional (3D) blue native (BN) polyacrylamide gel electrophoresis (PAGE) with protein identification by MS, BN northern blotting and functional analyses. Using this approach, the composition of the three major complexes could be elucidated and allowed a more detailed characterization of their potential functions.

## Materials and Methods

### Plant material and growth conditions

*Brassica napus* L. plants (cv Drakkar, provided by CBGP, Madrid, Spain) were grown on soil (3 : 1, soil to sand) in a glasshouse. The plants were cultivated in 30 cm pots in soil (Einheitserde Classic, Einheitserdewerk Uetersen, Germany) under controlled conditions (16 h : 8 h, light : dark, 22°C and 55% relative air humidity). Plants were watered automatically and additionally fertilized with Plantosan (Manna, Germany).

### Phloem sap sampling and protein separation

Phloem sap of *B. napus* was sampled as described previously (Giavalisco *et al*., 2006; Mendoza‐Cozatl *et al*., 2008). Hence, the inflorescence stems of 8‐wk‐old *B. napus* plants were punctured with a sterile hypodermic needle several times. After discarding the first visible droplets the subsequent exudate was collected into a precooled reaction tube. The purity of phloem was tested by reverse transcriptase PCR (RT‐PCR) with isolated mRNA from phloem sap and inflorescence stem tissue as described previously (Giavalisco *et al*., 2006). In total, 150 ng of RNA was used for cDNA synthesis using the RevertAid RT reverse transcription kit according to the manufacturer's protocol (ThermoFisher Scientific, Darmstadt, Germany). Five microlitres of the transcription samples was used for PCR using primers designed for Rubisco small subunit (forward: TTCACCGGCTTGAAGTCATC, reverse: CCGGGTACTCCTTCTTGCAT) and thioredoxin h (forward: CTCAAGGCAGCCAAAGAATC, reverse: ATGGC CTGAACATCGAACTC).

To separate the protein complexes of *B. napus* phloem sap a BN‐PAGE was performed as the first dimension (Wittig *et al*., 2006; Fiala *et al*., 2011). The phloem sap was first concentrated using a Vivaspin^®^ 500 centrifugal concentrator (MWCO 10 kDa; Sartorius, Göttingen, Germany) until the volume was one‐sixth of the start volume (6× phloem sap). The sample was then loaded onto a Novex NativePAGE 4–16% Bis‐Tris protein gel (Invitrogen) and electrophoresis was carried out at 150 V and 4°C with cathode buffer B (15 mM Bis‐Tris pH 7.0, 50 mM Tricine, 0.02% (w/v) Coomassie blue G250) and anode buffer (50 mM Bis‐Tris pH 7.0) in a Novex Gel System (Invitrogen). After the sample had passed one‐third of the gel the cathode buffer B was changed against cathode buffer B/10 (15 mM Bis‐Tris pH 7.0, 50 mM Tricine, 0.002% (w/v) Coomassie blue G250) and the separation was allowed to continue for 2–3 h at 150 V. Visible gel bands were excised and used for further analysis. For the subsequent denaturing gels, several phloem samples were run in parallel and gel pieces containing the same complex were combined and loaded into the same well of the second dimension gel.

For the Tris‐Tricine‐urea PAGE, which was performed as a second dimension, the excised gel pieces were incubated in 2× sodium dodecyl sulphate (SDS) loading buffer (Laemmli, 1970) for 10 min at room temperature, briefly boiled in a microwave and again incubated at room temperature for a further 15 min. The gel pieces were thentransferred onto the wells of a 10% Tris‐Tricine‐urea gel (thickness 1 mm) and the proteins were separated in a Mini‐PROTEAN^®^ 3 system (Bio‐Rad) at 150 V for 50 min. For the final SDS‐PAGE the gel lanes of interest were excised, first incubated in acidic buffer (100 mM Tris with 100 mM acidic acid) for 20 min and then equilibrated in 125 mM Tris‐HCl pH 6.8.

The equilibrated gel lanes were transferred onto the SDS gel (15%, thickness 1.5 mm) and covered with 0.5% (w/v) agarose in 1× SDS running buffer containing a trace of bromophenol blue. After protein separation at 150 V for 60 min, the polyacrylamide gels were stained with colloidal Coomassie (Dyballa & Metzger, 2009).

### Proteasome activity assay

The activity of the 20S proteasome was analysed using the Chemicon 20S Proteasome Activity Assay kit (APT280; EMD Millipore) following the instructions of the manufacturer. The assay is based on the detection of the fluorophore AMC being released from the substrate LLVY‐AMC after proteasomal cleavage. In brief, 10 μl of 6× phloem sap was added to 10 μl of 10× assay buffer and finally diluted to 90 μl with H_2_O. Then, 10 μl of the supplied substrate was added and fluorescence was measured in a Mithras 2 microplate reader equipped with a 380 : 460 nm filter set for 4 h at 37°C. Proteasome inhibition was achieved by adding 1 μl of the supplied irreversible inhibitor lactacystin to the test sample before measurement.

### Northern blot assay

To detect RNAs being part of the identified protein complexes, northern hybridization assays were performed as previously described (Yoo *et al*., 2004). The BN‐PAGE gels were transferred (Fastblot 43B semi dry Blot; Biometra, Göttingen, Germany) onto a H‐bond nylon membrane (Amersham Hybond‐N+, GE Healthcare Life Sciences, Uppsala, Sweden), UV‐crosslinked (Stratalinker 2400, Agilent, Santa Clara, CA, USA) and prehybridized with ULTRAhyb^©^ Ultrasensitive Hybridization Buffer (Ambion^®^; Life Technologies) for 1 h at 68°C. Then, 3′ biotinylated oligonucleotide probes (Supporting Information Table S1) were added and the membrane was incubated again at 68°C cooling down to 37°C for 12 h, before washing with 2× sodium saline citrate buffer (300 mM NaCl, 30 mM Na_3_citrate pH 7.0) at room temperature for 2 min. RNAs were detected using Pierce Chemiluminescent Nucleic Acid Detection Module Kit (ThermoFisher Scientific).

### Western blot assay

In order to detect ubiquitinated phloem proteins 6× phloem sap was incubated at 22°C for up to 4 h with and without the addition of lactacystin (Enzo Life Sciences, Lörrach, Germany). The proteins were separated by SDS‐PAGE on an 8% acrylamide gel and blotted onto a polyvinylidene difluoride (PVDF) membrane (0.45 μm, Immobilon‐P, EMD Millipore). The anti‐ubiquitin monoclonal antibody (BML‐PW8805, Enzo Life Sciences) and the secondary horseradish peroxidase (HRP)‐conjugated goat anti‐mouse (Dianova, Hamburg, Germany) were diluted 1 : 1000 and 1 : 100 000, respectively, in PBST (0.1% (v/v) Tween 20 in 1 × PBS (1 × phosphate buffered saline: 137 mM NaCl, 10 mM Na_2_HPO_4_, 1.8 mM KH_2_PO_4_, 2.7 mM KCl, pH 7.4)) with 2% skimmed milk powder. The detection was done using Pierce™ ECL western blotting substrate (ThermoFisher Scientific) and the ChemiDoc Touch Imaging System (Bio‐Rad).

### Mass spectrometry analysis

The stained protein spots were excised and transferred to siliconized 0.5 ml reaction tubes (Eppendorf, Hamburg, Germany). In‐gel digestion was performed according to Walz *et al*. (2002) with the following modifications. After de‐staining the gel pieces for 1 h in 50% (v/v) acetonitrile and 50% (v/v) 50 mM ammonium bicarbonate (NH_4_HCO_3_), the gel slices were dehydrated by adding 100% acetonitrile and incubation at ambient temperature for 10 min. After removing the acetonitrile the trypsin digestion was carried out overnight at 37°C with 0.01 μg μl^−1^ modified porcine trypsin (Promega) in 50 mM NH_4_HCO_3_. The supernatant was directly used for matrix‐assisted laser desorption/ionization (MALDI)‐MS analysis.

Sample and matrix preparation for AnchorChip™ Targets (Bruker Daltonics GmbH, Bremen, Germany) were performed according to the manufacturer's instructions and the acquisition of spectra was done on a Bruker Ultraflex III MALDI time‐of‐flight (TOF)/TOF mass spectrometer (Bruker Daltonics GmbH) operated in reflector mode. Positively charged ions were analysed in the *m*/*z* range 600–4000 Da. Monoisotopic signals were annotated with mMass (Strohalm *et al*., 2008) and protein identification was carried out using the Mascot data search engine (http://www.matrixscience.com) (Perkins *et al*., 1999) with the following search settings: 0.3 Da peptide tolerance, one permitted miscleavage and methionine oxidation as a variable modification. Searches were performed against the nonredundant database from NCBI (Pruitt *et al*., 2005) and were limited to *Viridiplantae*. Proteins were considered as identified if protein coverage of >25% and a significant Mascot score were reached. Protein hits with unknown function were blasted against the UniProtKB protein database (UniProt Consortium, 2014) to find homologous proteins with known function.

If more than one protein was identified in one spot by MALDI‐MS, MS/MS ion search on selected peptides was performed to confirm the additional proteins. Single peptides were set as parent ions and fragmentation was either achieved by laser or by additionally enabling collision‐induced decay (CID) using nitrogen as collision gas. Fragments were analysed with mMass using MS/MS ion search. Precursor peptide tolerance and MS/MS tolerance were set to 0.3. All further settings were equal to the peptide mass fingerprint analyses. A protein was regarded as identified if at least three peptides were assignable.

## Results

### Several native high‐molecular‐weight complexes exist in phloem sap

**Figure 1:**
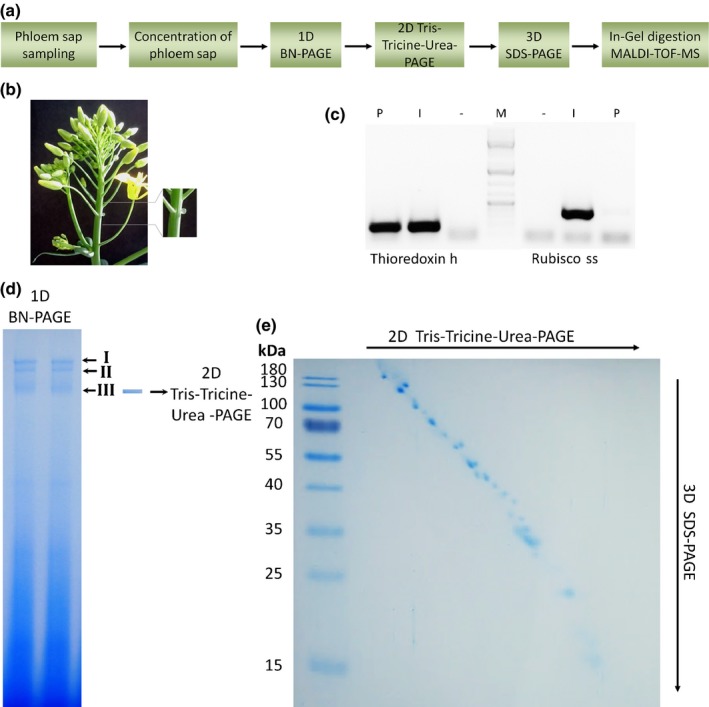
Overview of the procedure used to collect and analyse complexes from oilseed rape phloem sap exemplified on complex III. Schematic representation of the preparation steps to analyse complexes in phloem sap of *Brassica napus* (a). Eight‐week‐old plants were punctured at the inflorescence stem and, after removal of the first visible fluid, exuding phloem sap was collected and transferred into an ice‐cold reaction tube (b). The quality of phloem samples was verified by RT‐PCR (c) with RNA isolated from phloem sap (P) and the surrounding inflorescence stem extract (I). The presence of thioredoxin h (left) and Rubisco small subunit (right) mRNA was tested. To validate that the RT products result from RNA and not DNA, a negative control without reverse transcriptase was performed (–). The correct band size was evaluated against an additionally loaded DNA ladder as marker (M) (GeneRuler 1 kb plus; ThermoFisher Scientific). After concentrating the phloem sap to one‐sixth of the initial volume, 25 μl of the pure phloem sap was loaded per well onto a native gradient gel (4–16%) and protein complexes were separated by a BN‐PAGE. Visible bands of high‐molecular‐weight complexes (I–III) were cut out and each complex was transferred separately onto a denaturing 10% gel and separated by Tris‐Tricine‐PAGE (d). Finally the gel lanes of the second dimension were transferred onto a 15% gel and separated by SDS‐PAGE (e), before the distinct protein spots were visualized by colloidal Coomassie and prepared for MALDI‐TOF MS. Note: BN‐PAGE separates protein complexes in the megadalton range, whereas SDS‐PAGE separates single proteins up to *c*. 200 kDa.

Phloem sap from *B. napus* was sampled (Fig. 1b) as described previously (Giavalisco *et al*., 2006; Mendoza‐Cozatl *et al*., 2008), concentrated, and subjected to different combinations of native and denaturing gel electrophoreses, before proteins were identified by MALDI MS (the strategy is depicted in Fig. 1a). RT‐PCR analysis using primers against thioredoxin h and Rubisco small chain transcripts (Giavalisco *et al*., 2006; Mendoza‐Cozatl *et al*., 2008) ensured that RNA was present and that phloem samples were not contaminated by the content of tissue injured during sampling (Fig. 1c).

After initial separation by BN‐PAGE, several high‐molecular‐weight complexes were clearly visible (Fig. 1d). To confirm the existence of protein complexes, a second dimension denaturing SDS‐PAGE was run. Here, complexes are characterized by a specific pattern of bands in vertical orientation, while monomeric proteins show a hyperbolic curve (Swamy *et al*., 2006). To exclude artefacts, one sample was boiled for 5 min at 95°C before loading onto the native gel, which led to complete dissimilation of complexes (Fig. S1c,d).

**Figure 2:**
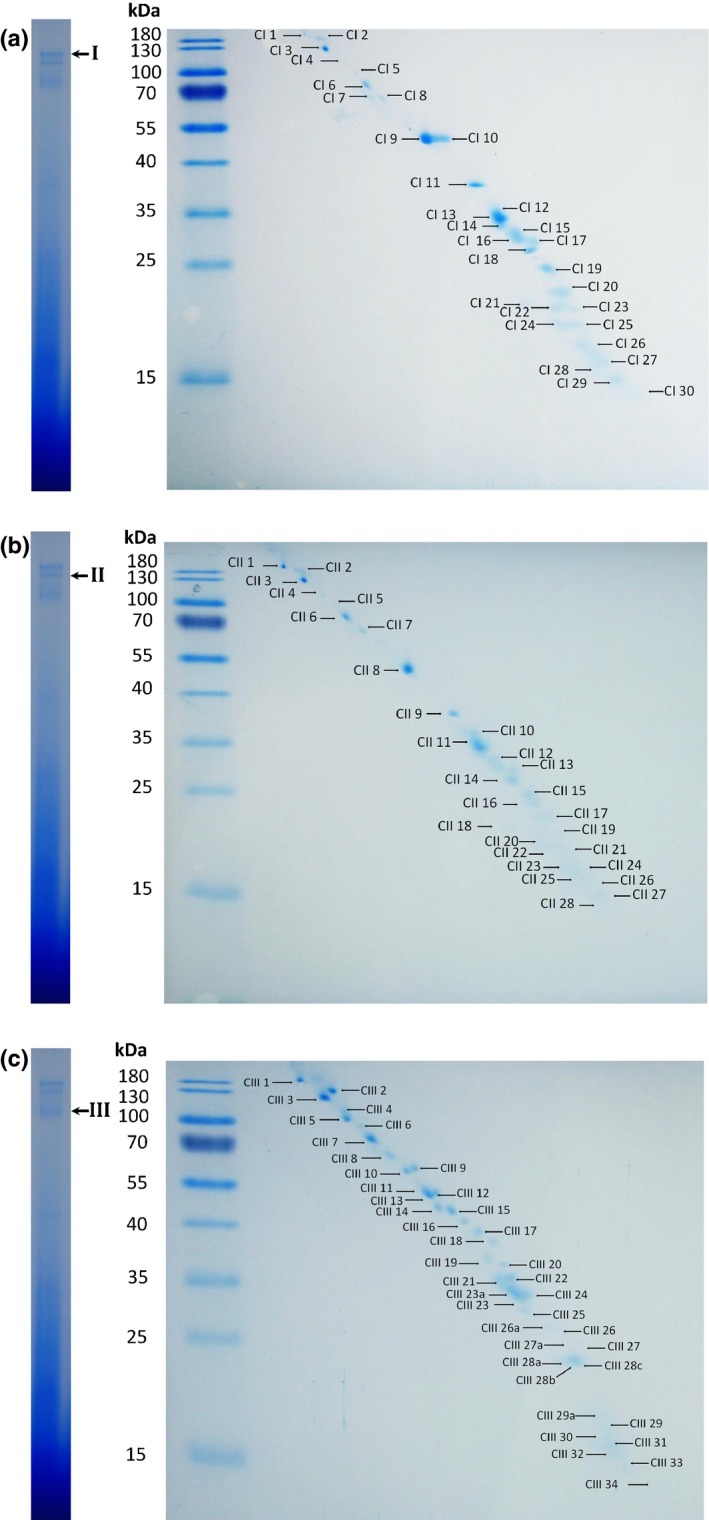
Third dimension of the three high‐molecular‐weight phloem complexes. The final separation of proteins contained in complexes I (a), II (b) and III (c) was performed by SDS‐PAGE in 15% gels. After separation, proteins were stained with colloidal Coomassie and the visible spots were in‐gel digested and identified by MALDI‐TOF MS.

To separate and identify single protein complex components, we followed a 3D gel electrophoresis approach. Here, single bands observed in the BN gel were excised and two denaturing PAGE steps were performed. For the first run a Tris‐Tricine‐urea PAGE was performed and a complete gel lane was then transferred onto a standard Laemmli SDS Tris‐glycine gel. This 3D PAGE allows a better separation of the proteins according to their hydrophobicity (Rais *et al*., 2004). Using this approach also proteins with very similar migration behaviour could be separated and concentrated in clear spots (e.g. Fig. 2c, spots 9 and 10; Fig. 2a, spots 16 and 17), which significantly enhanced protein identification by MS.

In total, three clearly visible protein complexes from the BN gel with an estimated size larger than 1 MDa were further separated and analysed, although other less abundant complexes might occur. 3D electrophoresis resulted in up to 30 different visible protein spots from each complex (Fig. 2) that were identified by MALDI‐TOF MS (Tables S2–S4).

Most protein components of the three high‐molecular‐weight complexes could be clearly assigned to either ribosomal subunits or proteasomes. In addition, a few unrelated proteins co‐migrated with the complexes, especially myrosinases and myrosinase‐binding proteins (Tables S2–S4), proteins known to form large aggregates up to 1000 kDa in size (Eriksson *et al*., 2002).

### Phloem sap contains ribosomal fragments

Different ribosomal proteins and also rRNAs have been found in phloem sap from different species (Giavalisco *et al*., 2006; Buhtz *et al*., 2008; Lin *et al*., 2009; Zhang *et al*., 2009), but if complexes or even completely assembled ribosomes exist has as yet not been analysed. Our results show that the largest three complexes contained different sets of ribosomal proteins. Complex I mainly consisted of many different 60S proteins, but no 40S proteins (Table S2). In total, 43% of all ribosomal proteins of the large subunit were found in this complex. Similar results were obtained for complex II. Again mainly 60S ribosomal proteins (33% of all 60S proteins) could be identified. Additionally a small fraction of 40S proteins was present within this complex (16% of all 40S proteins; Table S3). Since this complex is significantly smaller than complex I where no 40S proteins could be found, this second band may represent a distinct ribosomal fragment consisting of 40S and 60S ribosomal proteins and possibly RNA. Complex III did not contain any 60S ribosomal proteins, but rather resembles a 40S ribosomal fragment (28% of all 40S proteins; Table S4).

To detect additional proteins necessary for functional ribosomes, complete phloem proteome analysis was performed by LC‐MS/MS (Methods S1). Here several further ribosomal proteins were detectable including four large subunit proteins (L24, L27, L29 and L37) and eight small subunit proteins (S5, S10, S12, S17, S18, S23–25) (Table S5). Interestingly, whole proteome analyses using LC‐MS/MS could not identify 14 ribosomal proteins that were found in the analysis of native complexes by MALDI‐TOF MS, emphasizing the complementarity of the two experimental approaches. Still, important protein components of ribosomes could not be identified by combining both techniques, indicating that these proteins are of very low abundance or completely missing.

**Figure 3:**
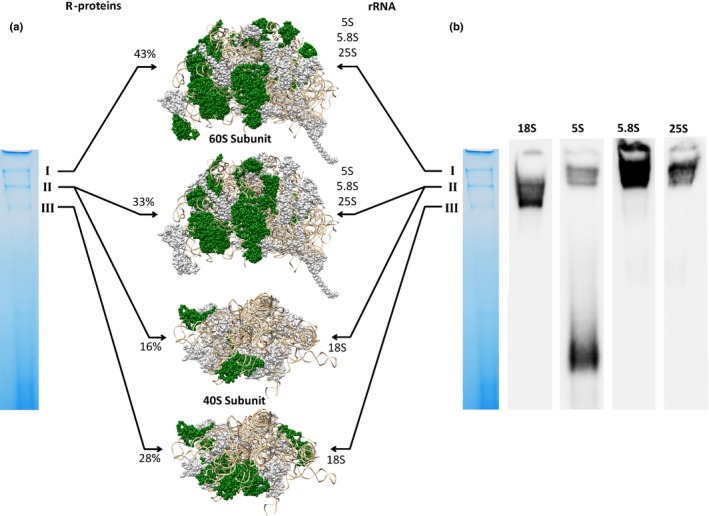
Phloem sap of *Brassica napus* contains ribosomal fragments consisting of r‐proteins and rRNAs. Depiction of r‐proteins identified by MALDI‐TOF MS analysis (highlighted in green in the 3D structure of the 60S and 40S subunit of *Saccharomyces cerevisiae* (pdb code: 4V7R)) (a). In total, 43% of all 60S r‐proteins were found in complex I. No 60S proteins were identified in complex III, while it contained 28% of all 40S r‐proteins. Complex II contained a mixture of 60S (33%) and 40S (16%) proteins. BN northern blot assays validated the presence of different rRNAs in the complexes (b).

Next we wanted to check whether the complexes contained rRNA additional to ribosomal proteins. To confirm the occurrence of native protein–RNA complexes, BN‐PAGE gels were directly blotted onto a nylon membrane and northern blots using probes characteristic for the three rRNAs of the large (25S, 5.8S and 5S) and for the 18S small subunit were performed. All three ribosomal complexes also showed distinct signals specific for the different rRNAs (Fig. 3b). Complex I representing a 60S fragment contained only large subunit rRNAs (25S, 5.8S and 5S), whereas complex III that contained only 40S ribosomal proteins also only contained 18S rRNA. Here, an additional signal for the 5S rRNA was visible far below the protein complexes, suggesting the occurrence of free 5S rRNA (Fig. 3b).

### Phloem sap contains active proteasomes and ubiquitinated proteins

**Figure 4:**
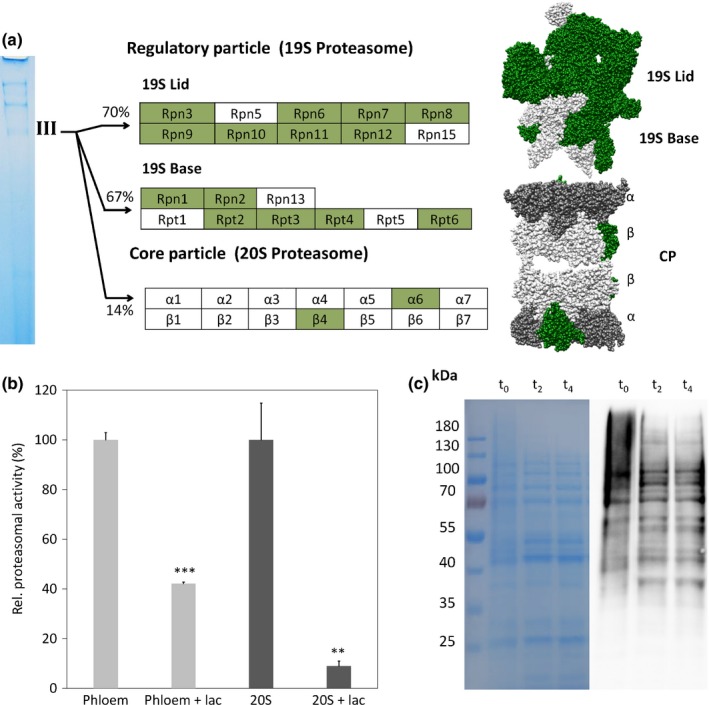
Phloem sap of oilseed rape contains an active proteasomal degradation pathway and ubiquitinated proteins. Overview of all proteasomal components and structure of the 19S–20S proteasome of *Saccharomyces cerevisiae* (pdb code: 5A5B). Proteins found in complex III by MALDI‐MS are highlighted in green. CP, core particle. (a). 20S proteasomal activity assays (b) were performed using native phloem sap (phloem) and as a control heat‐inactivated phloem sap supplemented with 20S proteasome positive control (20S). The addition of the proteasome‐specific inhibitor lactacystin (lac) significantly reduced the proteasomal activity in both samples. Errors bars represent + SD (phloem vs phloem+lac ***,* P *<* *0.001, *n *=* *5; 20S vs 20S+lac **, *P *<* *0.036, *n *=* *3). Western blot analysis for the detection of ubiquitinated proteins (c). Concentrated phloem sap (t_0_) was incubated at 22°C for 2 and 4 h (t_2_ and t_4_), separated by SDS‐PAGE (left) and transferred onto a PVDF membrane. Ubiquitinated proteins were detected using a primary antibody, which recognizes α‐mono‐, K^29^‐, K^48^‐ and K^63^‐linked polyubiquitinated proteins, and visualized by chemiluminescence using a secondary HRP‐conjugated antibody (right).

Recent proteome studies on denatured phloem proteins have shown the presence of proteasomal proteins in *Curcubita maxima* and *B. napus* phloem sap (Giavalisco *et al*., 2006; Lin *et al*., 2009), but the existence of assembled and functional proteasomes remained speculative. Besides 40S ribosomal proteins, proteasomal proteins co‐migrated with complex III (Table S4) and several regulatory as well as catalytical subunits were identified. Almost all regulatory proteins could be found (74%), including base and lid proteins of the 19S regulatory particle, but only two subunits (β‐4 and α‐6) from the core proteasome (20S proteasome) were detectable in the complex (14%; Fig. 4a; Table S4). Additional proteins could be identified by LC‐MS/MS analyses, finally covering 82% of all necessary proteasomal proteins (Table S6). To answer the question of whether this complex is a nonfunctional fragment or a proteolytically active proteasome, activity was tested using a 20S proteasome activity assay kit. To ensure that phloem sap is an environment suitable for proteasomal activity, phloem samples were boiled to inactivate enzymatic activities and pure 20S proteasome was added. A second sample was further treated with the proteasome‐specific inhibitor lactacystin. As clearly visible in Fig. 4(b), turnover of the proteasomal substrate LLVY‐AMC was possible in inactivated phloem samples and could be almost completely inhibited by lactacystin. In contrast to heat‐inactivated phloem sap, native phloem sap clearly degraded the proteasome‐specific substrate. Adding lactacystin to native phloem samples again led to a significant reduction of proteasome activity, although the loss of activity was minor compared with the positive control (Fig. 4b). These results suggest that despite the occurrence of several protease inhibitors in phloem sap (Giavalisco *et al*., 2006; Kehr, 2006) a proteasomal degradation pathway might be fully active in the phloem system. This is further supported by the finding of different proteins necessary for ubiquitination, including activating enzymes, ligases and deubiquitinating enzymes in other phloem proteome studies (Giavalisco *et al*., 2006; Lin *et al*., 2009; Gutierrez‐Carbonell *et al*., 2014).

It has also long been known that ubiquitin occurs in diverse phloem samples (Schobert *et al*., 1995). We additionally tested if there are any ubiquitinated phloem proteins using an antibody that detects α‐mono‐, K^29^‐, K^48^‐ and K^63^‐linked polyubiquitinated proteins. As visible in Fig. 4(c), many proteins seem to have multiple ubiquitin attachments. In particular, high‐molecular‐weight proteins were highly ubiquitinated. While total protein staining resulted in weak protein signals above 75 kDa, western blots showed intense signals in this region.

To determine whether ubiquitin‐conjugated proteins are subject to degradation by the endogenous phloem proteasomal pathway, phloem sap was incubated from 0 to 4 h at room temperature before SDS‐PAGE and western blotting. As visible in Fig. 4(c), ubiquitin signals decreased considerably after 2 and 4 h of incubation. Intriguingly the strongest decrease was observed in the high‐molecular‐weight range, since the smear observed in freshly denatured phloem sap decreased strongly with increasing incubation time. Moreover, several clear bands, especially below 75 kDa, remained unchanged (Fig. 4c), indicating that not all ubiquitin‐linked phloem proteins are targeted for proteasomal degradation.

## Discussion

Recent research on the phloem proteomes from different species led to the identification of a large number of phloem sap proteins and provided valuable insights into possible functional pathways present in this specialized compartment (Walz *et al*., 2002, 2004; Barnes *et al*., 2004; Giavalisco *et al*., 2006; Kehr, 2006; Lin *et al*., 2009; Rodriguez‐Medina *et al*., 2011). Not only proteins, but also RNAs, have been found including transcripts, tRNA halves, small RNAs and rRNAs (Kehr & Buhtz, 2013). The finding of several components of ribosomes (proteins and RNA) and proteasomes (proteins) in phloem sap led to the suggestion that protein synthesis and turnover might occur within the sieve tube system (Lin *et al*., 2009), but neither the existence of the relevant protein–protein or ribonucleoprotein complexes in phloem samples nor their activity could be demonstrated using conventional denaturing proteomic approaches.

In this study we were able to show that phloem samples contain several distinct high‐molecular‐weight complexes. Combining BN‐PAGE with two denaturing PAGE steps and MALDI‐TOF MS allowed the characterization of the three major complexes (Fig. 1d) in more detail. While BN‐PAGE allows the separation of intact protein complexes, the two denaturing PAGE steps (Rais *et al*., 2004) resulted in less co‐migration and hence an improved separation of the proteins contained in each of the complexes compared to 2D BN‐PAGE – SDS‐PAGE (Fig. 2 vs Fig. S1). The better protein separation led to improved identification with MS.

From each of the complexes, *c*. 30 components could be separated and most of the proteins were identified. All three complexes contained a set of ribosomal proteins. While the largest complex I contained proteins belonging to the 60S subunit, components of the 60S and the 40S subunits were present in complex II, while complex III showed only 40S proteins (Fig. 3; Tables S2–S4). Accordingly, direct BN northern blotting indicated that complex I and complex II contained 5S, 5.8 S and 25S rRNA, complex II additional 18S rRNA, and complex III only 18S rRNA (Fig. 3). This innovative combination of multi‐dimensional BN‐PAGE with BN northern blotting has great potential to analyse and characterize not only protein–protein but also ribonucleoprotein complexes.

The finding of three ribosomal fragments complexed with rRNAs again raises the question of the existence of complete ribosomes and translational activity in the angiosperm phloem (Lin *et al*., 2009). In this respect it is noteworthy that complete 80S ribosomes could probably not be separated by the approach applied here, caused by the size limitation of BN‐PAGE (according to the manufacturer's information). It is also difficult to assess the sizes of the observed complexes I–III, since it can be difficult to deduce reliable molecular masses of bands from their electrophoretic mobility in BN‐PAGE (Schamel, 2001; Eubel *et al*., 2005). This can be expected to apply even more so to ribonucleoprotein complexes. The isolation and separation of native ribonucleoprotein complexes in the present study increased the number of identified ribosomal proteins as compared to earlier proteome studies of denatured phloem samples (Giavalisco *et al*., 2006; Lin *et al*., 2009). However, a large fraction of ribosomal proteins (Fig. 3) or translation factors important for ribosomal function is still missing. In addition, a recent study addressing the question of translational activity in the phloem demonstrated that the phloem environment inhibits protein synthesis (Zhang *et al*., 2009). Taken together these observations indicate that the observed ribosomal fragments might be remnants from differentiating phloem cells or might only assemble to active complexes when released from the phloem environment, such as when the plant is wounded (Knoblauch & van Bel, 1998).

In contrast to ribosomal proteins, almost the entire proteasome and proteasome‐related proteins were discovered in an earlier study in *C. maxima* (Lin *et al*., 2009). In the present study in *B. napus* we could additionally show that many of the proteasome proteins indeed occur in a native protein complex (Fig. 4). The identified proteasome complex contains almost all regulatory proteins but fewer active proteins from the core particle could be identified. Furthermore, it could not be differentiated if a double‐capped or single‐capped proteasome is present or if the 19S regulatory and 20S core subunits co‐migrate in one band. By including information from LC‐MS/MS almost all proteasome subunits could be covered (Table S6). Also in this case we wanted to answer whether an active proteasomal degradation pathway exists is the phloem. It is known that proteasomes show trypsin‐like, chymotrypsin‐like and peptidylglutamyl protease activities (Voges *et al*., 1999). A proteasome activity assay indeed confirmed proteasomal degradation in phloem samples that could be inhibited by heat inactivation and the proteasome‐specific inhibitor for the chymotrypsin‐like activity lactacystin (Fig. 4b). Interestingly several protease inhibitors could be identified in recent studies and it was proposed that these inhibitors might act as a defence mechanism against phloem‐feeding insects (Kehr, 2006). They target different protease classes such as cysteine proteases, aspartyl proteases and serine proteases, but proteasome‐specific inhibitors seem to be absent.

These findings indicate that proteasomal degradation could actively degrade phloem proteins, given that ubiquitinated phloem proteins occur. To prove this, we analysed phloem sap samples in western blots using a ubiquitin‐specific antibody. Surprisingly a large fraction of phloem proteins in the whole size range seem to be ubiquitinated (Fig. 4c). The antibody used in this study detects mono‐, multimono‐ as well as polyubiquitinated proteins with isopeptide bonds at residues K^29^, K^48^ and K^63^. These different ubiquitin modifications fulfil different functions in plants. Mono‐ and multimono‐ubiquitination are related to proteolytic as well as nonproteolytic pathways such as endocytosis, nuclear transport and endosomal sorting (Hicke, 2001). Polyubiquitination events with K^48^ and K^29^ linkages are known to mark target proteins for degradation by the 26S proteasome (Chau *et al*., 1989; Johnson *et al*., 1995). K^63^ plays a role in intracellular signalling and DNA repair (Deng *et al*., 2000; Sobhian *et al*., 2007; Bennett & Harper, 2008; Grice & Nathan, 2016). The observation of protein ubiquitination in phloem samples over time showed that especially proteins larger than 75 kDa lost their ubiquitin modification, while the pattern of smaller proteins remained nearly unchanged (Fig. 4c). This indicates that not all ubiquitinated phloem proteins are potential targets for proteasomal degradation and that ubiquitination of phloem proteins might have additional functions, for example in protein sorting. On the other hand, phloem proteasomes might have functions additional to phloem protein degradation. They could, for example, be involved in defence responses by marking and degrading pathogen proteins, since the phloem is a favourite target for intruding pathogens such as viruses and bacteria. Many viruses use the phloem long‐distance translocation system to spread throughout the whole plant and are transmitted by phloem‐feeding insects (Hipper *et al*., 2013). In this context it was suggested previously that proteasome‐mediated proteolysis could play a role in restricting viral proteins to the phloem (Mekuria *et al*., 2008) and that proteasomes might be involved in virus degradation (Hipper *et al*., 2013).

Taken together, the analysis of protein–protein and ribonucleoprotein complexes by multidimensional BN‐PAGE performed in the present study proved the presence of complexes in native phloem samples. Additional activity tests further substantiated that active proteasomes exist in phloem samples, while translational activity is questionable, although ribosome fragments consisting of proteins and RNA occur. Our results therefore complement and extend earlier findings of single components of these complexes and substantiate speculations about potential translational and proteasomal activities. The surprisingly large number of ubiquitinated phloem proteins and their observed stability despite proteasome activity suggests specialized roles for ubiquitin modifications of endogenous phloem proteins, as well as additional, probably external, targets for proteolytic degradation in the phloem. Finally, the direct co‐detection of RNA in ribonucleoprotein complexes by BN northern blotting introduced in the present study opens a range of new possibilities for the analysis of endogenous ribonucleoprotein complexes and for systematic protein–RNA interaction studies.

## Author contributions

A.O. and S.P. designed the experiments and performed phloem sap sampling, BN‐PAGE, MALDI‐TOF MS, proteasome activity assay, western blots, analysed the data and participated in writing the manuscript. L.K. performed northern blots, and P.H. performed phloem sap sampling for LC‐MS/MS. M.Y.G. and J.D. performed phloem sap sampling and BN‐PAGE. P.G. performed LC‐MS/MS and analysed the data, J.K. conceived the study and wrote the manuscript.

## Supporting information

Please note: Wiley Blackwell are not responsible for the content or functionality of any Supporting Information supplied by the authors. Any queries (other than missing material) should be directed to the New Phytologist Central Office.

**Fig. S1** Overview of a 2D gel electrophoresis approach for the identification of protein complexes in oilseed rape phloem sap.
**Table S1** Oligonucleotides used to detect rRNAs in the phloem sap of *Brassica napus*
**Table S2** List of proteins identified by MALDI‐TOF MS from complex I corresponding to the spot numbers in Fig. 2(a)
**Table S3** List of proteins identified by MALDI‐TOF MS from complex II corresponding to the spot numbers in Fig. 2(b)
**Table S4** List of proteins identified by MALDI‐TOF MS from complex III corresponding to the spot numbers in Fig. 2(c)
**Table S5** Ribosomal proteins identified with LC‐MS/MS from whole, denatured phloem samples.
**Table S6** Proteasomal proteins identified with LC‐MS/MS from whole, denatured phloem samples.
**Methods S1** Analysis of phloem proteome samples by LC‐MS/MS.
