# Supplementary material for: Functional analysis of Brassica napus phloem protein and ribonucleoprotein complexes
Source: New Phytol. 2017 Jan 4;214(3):1188–97. doi: 10.1111/nph.14405 (PMC6079638; doi:10.1111/nph.14405)
Supplement: Supplementary file 1 — Fig. S1 Overview of a 2D gel electrophoresis approach for the identification of protein complexes in oilseed rape phloem sap. Table S1 Oligonucleotides used to detect rRNAs in the phloem sap of Brassica napus Table S2 List of proteins identified by MALDI‐TOF MS from complex I corresponding to the spot numbers in Fig. 2(a) Table S3 List of proteins identified by MALDI‐TOF MS from complex II corresponding to the spot numbers in Fig. 2(b) Table S4 List of proteins identified by MALDI‐TOF MS from complex III corresponding to the spot numbers in Fig. 2(c) Table S5 Ribosomal proteins identified with LC‐MS/MS from whole, denatured phloem samples. Table S6 Proteasomal proteins identified with LC‐MS/MS from whole, denatured phloem samples. Methods S1 Analysis of phloem proteome samples by LC‐MS/MS. [file NPH-214-1188-s001.pdf]

New Phytologist

New Phytologist Supporting Information Fig. S1, Tables S1-S6 and Methods S1

Article title: Functional analysis of *Brassica napus* phloem protein and ribonucleoprotein complexes

Authors: Anna Ostendorp, Steffen Pahlow, Lena Krüssel, Patrizia Hanhart, Marcel Y. Garbe, Jennifer Deke, Patrick Giavalisco and Julia Kehr

Article acceptance date: 26 November 2016

The following Supporting Information is available for this article:

Fig. S1 Overview of a 2D gel electrophoresis approach for the identification of protein complexes in oilseed rape phloem sap

Table S1 Oligonucleotides used to detect ribosomal RNAs in the phloem sap of *B. napus*

Table S2 List of proteins identified by MALDI-TOF MS from complex I corresponding to the spot numbers in Fig. 2(a)

Table S3 List of proteins identified by MALDI-TOF MS from complex II corresponding to the spot numbers in Fig. 2(b)

Table S4 List of proteins identified by MALDI-TOF MS from complex III corresponding to the spot numbers in Fig. 2(c)

Table S5 Ribosomal proteins identified with LC-MS/MS from whole, denatured phloem samples

Table S6 Proteasomal proteins identified with LC-MS/MS from whole, denatured phloem samples

Methods S1 Analysis of phloem sap proteins by LC-MS/MS

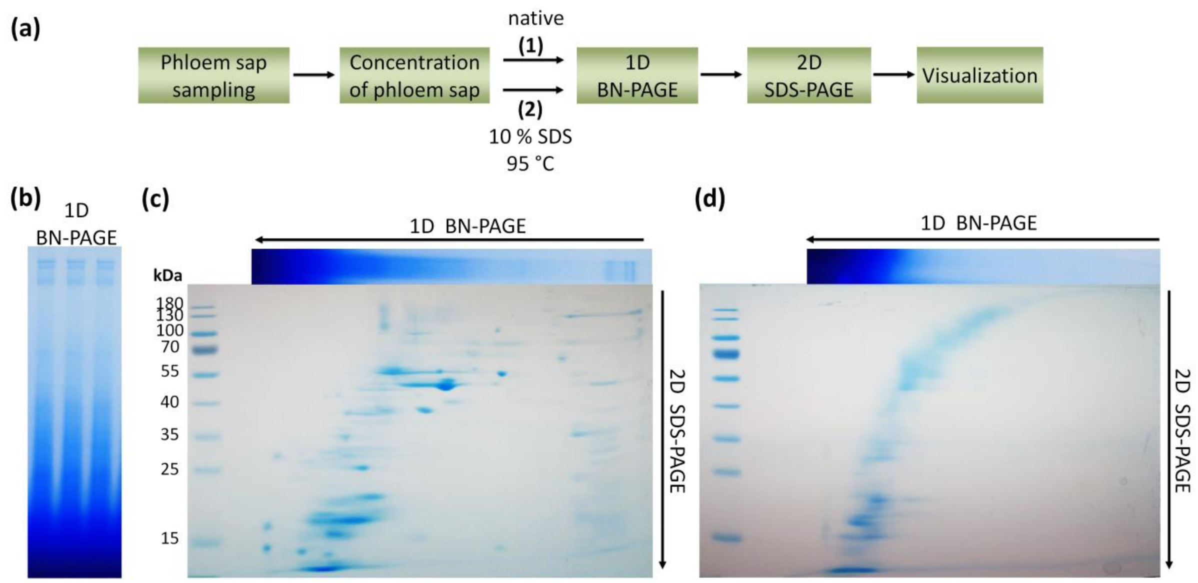

Fig. S1 Overview of a 2D gel electrophoresis approach for the identification of protein complexes in oilseed rape phloem sap**.** Flow diagram of the working procedure to separate multiprotein complexes (a). For the first identification of multiprotein complexes in the phloem translocation stream 6x concentrated phloem sap was separated under native conditions by BN-PAGE for the first dimension (b). For the second dimension the proteins were treated with SDS and finally separated under denaturing conditions by SDS-PAGE. While monomeric proteins of denatured phloem sap (treated with 10% SDS and 95°C for 10 min) migrate in a hyperbolic diagonal (d), components of multiprotein complexes of native phloem sap were located in a vertical line below the hyperbolic diagonal (c).

New Phytologist

Table S1 Oligonucleotides used to detect ribosomal RNAs in the phloem sap of *B. napus*

rRNA	Organism	Accession no.[a]	Sequence	Target sequence[b]
5S	*B. napus*	gi|17866	AAGCACGCTTAACTGCGGAGTTCTGAT	5S rRNA sense (37…63)
5.8S	*B. napus*	gi|217882:195-358	CGATGGTTCACGGGATTCTGCAATTCA	5.8S rRNA sense (72…99)
25S	*B. napus*	gi|217882:546-3923	CCTGATGCGGTTATGAGTACGACCG	25S rRNA sense (18…42)
18S	*B. napus*	CCCW010000853	CAAGAAAGAGCTCTCAGTCTGTCAATC	18S rRNA sense (1218…1247)

[a b]Accession no. and target sequence are from NCBI database or from SILVA ribosomal RNA gene database (Pruesse *et al.*, 2007; Quast *et al.*, 2013; Yilmaz *et al.*, 2014).

Table S2 List of proteins identified by MALDI-TOF MS from complex I corresponding to the spot numbers in Fig. 2(a)

Spot no.	MW obs. [kDa]	Annotation[a]	Organism	Accession no.[b]	MW [kDa]	MASCOT score
CI_1	180	n.i.				
CI_2	150	myrosinase-binding protein 2-like	B.n	XP_013655529	115.6	173
CI_3	120	myrosinase-binding protein 2-like	B.n	CDY51195	116.0	162
CI_4	100	n.i.				
CI_5	80	uncharacterized protein	B.n	CDY08190	90.0	90
CI_6	70	myrosinase-like isoform X2	B.n	CDY37226	61.8	130
CI_7	60	n.i.				
CI_8	60	myrosinase	B.n	ABQ42337	60.0	154
CI_9	45	60S ribosomal protein L4-1	B.n	CDY44721	44.6	91
		60S ribosomal protein L3-1	B.r	XP_009123677	44.6	90
CI_10	45	60S ribosomal protein L4-1	B.n	CDY44721	44.6	111
CI_11	38	60S ribosomal protein L5	B.n	CDY15089	39.1	103
CI_12	35	60S ribosomal protein L7Ae	B.n	CDX76668	32.2	91
		60S ribosomal protein L8-3-like	B.r	XP_009138409	28.0	83
CI_13	34	60S ribosomal protein L8-1-like	B.n	XP_013672357	28.0	109
CI_14	33	60S ribosomal protein L1	B.o	XP_013602047	26.2	110
CI_15	32	60S ribosomal protein L13-1-like	B.r	XP_009149775	23.6	117
CI_16	30	60S ribosomal protein L7-2	B.o	XP_013616063	27.9	134
CI_17	30	60S ribosomal protein L10a-2-like	B.r	XP_009103604	24.4	82
CI_18	28	60S ribosomal protein L15-1-like	B.n	XP_013739641	24.3	143
CI_19	24	60S ribosomal protein L9-1	B.n	CDY32946	21.9	96
CI_20	23	60S ribosomal protein L18a-2-like	B.r	XP_009132991	21.2	54*
CI_21	22	60S ribosomal protein L23a	B.n	CDX93259	24.3	82
		60S ribosomal protein L26-1-like	B.n	XP_013748571	16.9	96
CI_22	22	60S ribosomal protein L21-1	B.n	XP_013714937	18.6	57*
CI_23	22	60S ribosomal protein L11-2 isoform X1	B.r	XP_009132184	20.9	159
CI_24	20	60S ribosomal protein L23a-1-like	B.r	XP_009133367	16.9	102
		60S ribosomal protein L26-1-like	B.n	XP_013748571	16.9	98
CI_25	20	60S ribosomal protein L23a-2	B.n	XP_013691990	17.3	113
		60S ribosomal protein L12-2-like	B.r	XP_009116035	17.9	86
CI_26	18	60S ribosomal protein L18e	B.n	CDY12475	12.7	82
CI_27	16	60S ribosomal protein L34-2-like	B.r	XP_009127750	13.7	89
		60S ribosomal protein L35-1	B.n	CDY07930	18.0	81
CI_28	15	n.i.				
CI_29	14	60S ribosomal protein L14-2	B.o	XP_013583256	15.5	80
CI_30	13	60S acidic ribosomal protein P2-2-like	B.r	XP_009140817	11.4	47*
		60S ribosomal protein L30-2	B.r	XP_009106439	12.2	59*

[a]Annotations are from NCBI database or from BLAST-matched *Arabidopsis* annotations.
[b]Accession no. are from NCBI database only.
*Asterisk at not significant MASCOT score values indicates proteins identified by MS/MS ion search.
Abbreviations: n.i: not identified, MW: molecular weight, obs.: observed, B.n: *Brassica napus*, B.o: *Brassica oleracea*, B.r: *Brassica rapa*.

Table S3 List of proteins identified by MALDI-TOF MS from complex II corresponding to the spot numbers in Fig. 2(b)

Spot no.	MW obs. [kDa]	Annotation[a]	Organism	Accession no.[b]	MW [kDa]	MASCOT score
CII_1	190	clathrin heavy chain 1	B.r	XP_009146750	193.3	146
CII_2	120	myrosinase-binding protein 2-like	B.n	XP_013655529	115.6	117
CII_3	110	myrosinase-binding protein 2-like	B.n	XP_013724785	116.1	223
CII_4	100	n.i.				
CII_5	90	n.i.				
CII_6	75	myrosinase-like isoform X2	B.n	XP_013729265	61.9	105
CII_7	60	myrosinase-like precursor	B.n	XP_013678032	62.7	87
CII_8	49	60S ribosomal protein L3-1-like isoform X1	B.n	XP_013696748	45.4	87
CII_9	37	60S ribosomal protein L5	B.n	CDY15089	36.8	137
CII_10	36	n.i.				
CII_11	35	40S ribosomal protein S6-1-like	B.n	XP_013650213	28.4	93
CII_12	34	40S ribosomal protein S4-1-like isoform X1	B.o	XP_013620505	29.6	112
		60S ribosomal protein L8-3	B.r	XP_009138409	27.9	92
CII_13	33	60S ribosomal protein L13-1 or 2	B.r	XP_009151668	23.6	106
CII_14	32	60S ribosomal protein L10a-2-like	B.r	XP_009103604	24.5	82
CII_15	31	60S ribosomal protein L7-2	B.o	XP_013616063	27.9	93
CII_16	30	60S ribosomal protein L15-1-like	B.n	XP_013739641	24.2	90
CII_17	25	60S ribosomal protein L9-1-like	B.n	XP_013717489	22.0	84
CII_18	24	40S ribosomal protein S9-1	B.r	XP_009131450	22.9	127
		60S ribosomal protein L9-1	B.r	XP_009123738	22.0	83
CII_19	22	n.i				
CII_20	21	60S ribosomal protein L17-2	B.r	XP_009127555	19.8	124
CII_21	20	60S ribosomal protein L11	B.n	CDY62552	19.7	104
CII_22	19	60S ribosomal protein L21-1-like	B.r	XP_009110810	18.5	87
CII_23	18	40S ribosomal protein S15-1	B.r	XP_009118657	17.1	85
CII_24	18	60S ribosomal protein L23a-1	B.r	XP_009143312	17.4	109
CII_25	17	40S ribosomal protein S16-3-like	B.r	XP_009134755	16.5	123
CII_26	17	60S ribosomal protein L32-1-like	B.n	XP_013654096	15.2	79
CII_27	16	60S ribosomal protein L36-2-like	B.o	XP_013625552	12.6	108
CII_28	15	60S ribosomal protein L14-2-like	B.o	XP_013583256	15.5	141
		60S ribosomal protein L22-3	B.n	XP_013703751	14.0	100

[a]Annotations are from NCBI database or from BLAST-matched *Arabidopsis* annotations.
[b]Accession no. are from NCBI database only.
Abbreviations: n.i: not identified, MW: molecular weight, obs.: observed, B.n: *Brassica napus*, B.o: *Brassica oleracea*, B.r: *Brassica rapa*.

Table S4 List of proteins identified by MALDI-TOF MS from complex III corresponding to the spot numbers in Fig. 2(c)

Spot no.	MW obs. [kDa]	Annotation[a]	Organism	Accession no.[b]	MW [kDa]	MASCOT score
CIII_1	180	clathrin heavy chain 1-like	B.n	XP_013729579	193.3	93
CIII_2	130	26S proteasome regulatory complex component	B.n	CDY19162	108.9	108
CIII_3	125	myrosinase-binding protein 2-like	B.n	XP_013724785	116.1	226
CIII_4	110	26S proteasome regulatory complex component	B.n	CDY29907	97.8	90
CIII_5	100	26S proteasome non-ATPase regulatory subunit 2 homolog B	B.o	XP_013598567	97.7	83
CIII_6	95	n.i.				
CIII_7	70	myrosinase-like isoform X2	B.n	XP_013729265	61.9	116
CIII_8	65	myrosinase-like precursor	B.n	NP_001303128	62.6	94
CIII_9	60	Proteasome regulatory subunit C-terminal	B.n	CDX87648	54.8	82
CIII_10	58	26S proteasome regulatory subunit 4 homolog B-like	B.n	XP_013707211	45.8	142
CIII_11	55	n.i.				
CIII_12	50	26S protease regulatory subunit 6B-like protein	B.n	CDY32995	45.4	121
CIII_13	50	tRNA (guanine(10)-N2)-methyltransferase homolog	B.o	XP_013613398	54.1	118
CIII_14	45	26S protease regulatory subunit 8 homolog A-like	B.o	XP_013620664	47.1	88
CIII_15	45	26S protease regulatory subunit 10B homolog A<br>26S proteasome non-ATPase regulatory subunit 11 homolog	B.r<br>B.n	XP_009123705<br>CDY43320	44.7<br>46.6	121<br>86
CIII_16	40	26S proteasome non-ATPase regulatory subunit 6 homolog	B.r	XP_009109236	44.1	81
CIII_17	39	26S proteasome non-ATPase regulatory subunit 13 homolog	B.o	XP_013622244	43.9	86
CIII_18	38	26S proteasome non-ATPase regulatory subunit 7 homolog A	B.n	XP_013724003	34.7	91
CIII_19	37	guanine nucleotide-binding protein subunit beta-like	B.n	XP_013641806	35.7	141
CIII_20	36	26S proteasome non-ATPase regulatory subunit 14	B.n	CDY59032	26.5	92
CIII_21	35	ribosomal protein uS5	B.n	CDY06903	27.9	89
CIII_22	35	40S ribosomal protein S3-2-like	B.n	XP_013734128	27.3	102
CIII_23	30	n.i.				
CIII_23a	31	40S ribosomal protein S4-1-like	B.o	XP_013622723	29.7	112
CIII_24	30	40S ribosomal protein S8<br>40S ribosomal protein S4-1-like	B.n<br>B.n	CDX92472<br>XP_013708194	27.3<br>29.8	101<br>86
CIII_25	28	26S proteasome non-ATPase regulatory subunit 8 homolog A	B.o	XP_013608683	30.7	84
CIII_26a	27	proteasome subunit alpha type-6-B	B.r	XP_009102355	27.3	111
CIII_26	26	n.i.				
CIII_27a	25	n.i.				
CIII_27	24	proteasome subunit beta type-4	B.n	CDY39004	29.4	107
CIII_28a	22	40S ribosomal protein S7	B.n	XP_013735701	22.1	82
CIII_28b	22	40S ribosomal protein S9-1	B.r	XP_009131450	22.9	83

CIII_28c	22	n.i.				
CIII_29a	18	n.i.				
CIII_29	17	n.i.				
CIII_30	16	40S ribosomal protein S13-2 isoform X2	B.o	XP_013590122	15.9	89
CIII_31	15	n.i.				
CIII_32	14	n.i.				
CIII_33	13	40S ribosomal protein S19-1	B.r	XP_009147386	15.8	104
CIII_34	12	40S ribosomal protein S15a-1-like	B.n	XP_013652949	13.4	76*

[a]Annotations are from NCBI database or from BLAST-matched *Arabidopsis* annotations.
[b]Accession no. are from NCBI database only.
*Asterisk at not significant MASCOT score values indicates proteins identified by MS/MS ion search.
Abbreviations: n.i: not identified, MW: molecular weight, obs.: observed, B.n: *Brassica napus*, B.o: *Brassica oleracea*, B.r: *Brassica rapa*.

Table S5 Ribosomal proteins identified with LC-MS/MS from whole, denatured phloem samples

Accession no.[a]	Peptide count	No. unique peptides	Confidence score	Annotation[b]
A0A078C4Q2	6	2	172.86	BnaC08g30490D, ribosomal protein L37Ae
A0A078DKH7	13	2	426.71	BnaA01g14960D, ribosomal protein S10-1
A0A078H7X0	5	2	322.36	BnaA01g12190D, ribosomal protein L7Ae
A0A078IVX8; A0A078GIA9; A0A078JQ43	14	2	973.05	BnaCnng24330D, ribosomal protein L10P
A0A078CAT0	13	2	956.63	BnaA03g30850D, ribosomal protein L10P
A0A078CBY4	3	2	122.43	BnaA03g26700D, ribosomal protein L12
A0A078ESE5	10	2	216.78	BnaAnng00420D, ribosomal protein L29
A0A078D1R2	14	2	418.84	BnaA01g20600D, ribosomal protein S17
A0A078HL45	5	2	103.94	BnaA02g19870D, ribosomal protein L37e
A0A078DSU4	5	2	83.12	BnaA08g17260D, ribosomal protein L44e
A0A078F0G6	11	2	339.37	BnaC03g35930D, ribosomal protein L29
A0A078IW24; A0A078E102	12	2	368.89	BnaA05g34620D, ribosomal protein L23e
A0A078CJR7; A0A078E231	9	2	215.66	BnaC04g39150D, ribosomal protein L10
A0A078J2A2	10	2	450.93	BnaA09g51640D, ribosomal protein S24
A0A078BWX6	8	2	239.71	BnaA07g15380D, ribosomal protein S10
A0A078G4Q0; A0A078GKI4	5	2	151.6	BnaA09g29970D, ribosomal protein L27a-2
A0A078FXI3; A0A078CRJ6; A0A078D2T0; A0A078GDG3	19	2	484.93	BnaC04g03350D, ribosomal protein L7
A0A078DB19; A0A078EDI1; A0A078G0I6	6	2	159.93	BnaA06g31830D, ribosomal protein L12
A0A078I2A8	13	2	636	BnaA01g33230D, ribosomal protein S19
A0A078H7G9	6	2	112.22	BnaC05g12160D, ribosomal protein S12
A0A078FG63	3	2	88.76	BnaC04g12660D, ribosomal protein S12
A0A078D0U1	21	2	587.24	BnaC08g37220D, ribosomal protein L6
A0A078C209	20	2	633.44	BnaC01g03750D, ribosomal protein S3a
A0A078EL71	7	2	130.22	BnaC09g01930D, ribosomal protein L34-3
A0A078C6N9	21	2	663.69	BnaC07g45170D, ribosomal protein S3a
A0A078D6H5; A0A078CW86; A0A078IP49	18	2	529.12	BnaA03g14630D, ribosomal protein S3
A0A078DMB9	9	2	411.29	BnaC03g46180D, ribosomal protein S25
A0A078BZC2; A0A078J0L6	14	3	523.94	BnaA07g23080D, ribosomal protein SA
A0A078BT17; A0A078CHG5; A0A078CNK9; A0A078DUM4; A0A078IYP6	20	3	729.48	BnaA07g19460D, ribosomal protein L7a-1

A0A078CGP6	6	3	158.1	BnaA03g29090D, ribosomal protein L18-2
A0A078IBN0; A0A078D7X9	3	3	91.8	BnaA02g29320D, ribosomal protein P2-3
A0A078DPY8; A0A078DTE3; A0A078JAE5; P46289	12	3	527.71	BnaA03g42820D, ribosomal protein L15
A0A078DS49; A0A078CAM5	11	4	386.32	BnaC04g08010D, ribosomal protein S5-1
A0A078IT30; A0A078HAI1	14	4	545.35	BnaC01g41990D, ribosomal protein S7
A0A078F240	17	4	563.44	BnaC08g20700D, ribosomal protein L13
A0A078GTP4; A0A078EPL9; A0A078FS35	13	4	272.2	BnaC06g26740D, ribosomal protein L17-2
A0A078DKR3; A0A078CYS0	14	5	576.23	BnaA10g02510D, ribosomal protein S15-1
A0A078GXC1	20	5	657.88	BnaC01g19250D, ribosomal protein L14-2

[a] [b]Accession no. and annotations are from UniProtKB or from BLAST-matched *Arabidopsis* annotations.

New Phytologist

Table S6 Proteasomal proteins identified with LC-MS/MS from whole, denatured phloem samples

Accession no.[a]	Peptide count	No. unique peptides	Confidence score	Annotation[b]
A0A078FMY0	10	2	261.2	BnaA08g13700D, proteasomal protein Rpn1
A0A078CA44; A0A078CS49	14	2	703.74	BnaC08g22660D, proteasomal protein alpha-7
A0A078GC78	18	2	704.23	BnaA06g00680D, proteasomal protein alpha-1
A0A078ETP8	15	2	749.04	BnaC04g18160D, proteasomal protein beta-4
A0A078FU71; A0A078IN04	12	2	455.75	BnaA07g04410D, proteasomal protein alpha-6
A0A078DDU6	11	2	394.34	BnaA07g34730D, proteasomal protein alpha-2
A0A078BYM7; A0A078FFR2	11	2	477.84	BnaA07g20430D, proteasomal protein alpha-2
A0A078JIE4; A0A078G1G5	12	2	544.71	BnaAnng21900D, proteasomal protein alpha-4
A0A078EM15; A0A078HKE7; A0A078JJU3	9	3	410.78	BnaC07g14280D, proteasomla protein beta-3
A0A078HLZ9; A0A078C9Z6; A0A078HBR3	25	3	1142.17	BnaC01g09980D, proteasomal protein Rpn1
A0A078C7A7	6	3	225.27	BnaC07g47640D, proteasomal protein Rpn10
A0A078DI11; A0A078C3X6	39	3	1780.82	BnaA03g07840D, proteasomal protein Rpt6
A0A078D2A1; A0A078HMH1; A0A078JNL6	7	4	143.63	BnaA03g22660D, proteasomal protein Rpn13
A0A078DU50; A0A078JIY2	24	4	1086.07	BnaC07g41330D, proteasomal protein Rpn1
A0A078EB70; A0A078D1P0; A0A078FT37; A0A078G6L6	12	4	543.27	BnaA06g07650D, proteasomal protein beta-5
A0A078ETP2	10	5	138.13	BnaC02g36840D, proteasomal protein beta-7

[a] [b]Accession no. and annotations are from UniProtKB or from BLAST-matched *Arabidopsis* annotations.

Methods S1 Analysis of phloem sap proteins by LC-MS/MS

50 µg of extracted and precipitated phloem sap protein pellets were re-suspended in 200 µl of protein extraction buffer (6 M urea, 2 M thiourea, 15 mM DTT, 2% CHAPS). Once the proteins were dissolved, the samples were sonicated for 10 min in a sonication bath, followed by an additional 30 min incubation on an orbital shaker (100 rpm) at room temperature. Solubilized proteins were centrifuged at 10000 ***g*** for 5 min and the protein concentration was determined from the collected supernatant. 50 µg of proteins extract were digested in-solution using a Trypsin/Lys-C mixture (Mass Spec Grade, Promega, Madison, WI, USA) according to the instruction manual. After the digestion, the samples were desalted using C_{18}-stage tips as described in Rappsilber *et al.* (2007).
After the elution of the digested and desalted peptides from C_{18}-stage tips, the samples were concentrated to near dryness in a SpeedVac and the peptide mixtures were analyzed by LC-MS/MS using a Q ExactivePlus (Thermo Scientific, Bremen, Germany) high resolution mass spectrometer connected to an EASY-nLC 1000 system (Thermo Scientific) equipped with a 75 µm × 15 cm Acclaim PepMap RSLC HPLC column (Thermo Scientific). Peptides were separated using a binary buffer system of 0.1% formic acid in water (Buffer A) and 60% acetonitrile containing 0.1% formic (Buffer B). The flow rate was adjusted to 300 nl min^{-1}. Peptides were eluted with on a linear gradient of 0–40% buffer B for 50 min followed by a linear gradient between 40–80% buffer B for additional 30 min. Peptides were analyzed with one full scan (200–2000 m/z, R = 70,000 at 200 m/z), followed by up to fifteen data-dependent MS/MS scans (Top 15 approach) with higher-energy collisional dissociation (HCD) at a resolution of 17500 at 200 m/z. Dynamic exclusion was set to 30 s. Raw data were processed using the Progenesis QI for proteomics (Progenesis QI for Proteomics Version 3.0, Nonlinear Dynamics, Newcastle, UK) software in combination with the Mascot (Version 2.5, MatrixScience, Boston MS, USA) database search tool using the Arabidopsis TAIR database (Version 10, The Arabidopsis Information Resource, www.arabidopsis.org.).

References

Pruesse E, Quast C, Knittel K, Fuchs BM, Ludwig W, Peplies J, Glöckner FO. **2007**. SILVA: a comprehensive online resource for quality checked and aligned ribosomal RNA sequence data compatible with ARB. *Nucleic Acids Research* **35**: 7188–7196.

Quast C, Pruesse E, Yilmaz P, Gerken J, Schweer T, Yarza P, Peplies J, Glöckner FO. **2013**. The SILVA ribosomal RNA gene database project: improved data processing and web-based tools. *Nucleic Acids Research* **41**: D590–596.

Rappsilber J, Mann M, Ishihama Y. **2007**. Protocol for micro-purification, enrichment, pre-fractionation and storage of peptides for proteomics using StageTips. *Nature Protocols* **2**: 1896–1906.

Yilmaz P, Parfrey LW, Yarza P, Gerken J, Pruesse E, Quast C, Schweer T, Peplies J, Ludwig W, Glöckner FO. **2014**. The SILVA and 'All-species Living Tree Project (LTP)' taxonomic frameworks. *Nucleic Acids Research* **42**: D643–648.
